# Scalp Electroacupuncture Promotes Angiogenesis after Stroke in Rats by Activation of Wnt/*β*-Catenin Signal Pathway

**DOI:** 10.1155/2022/1649605

**Published:** 2022-03-14

**Authors:** Shuai Shi, Mingye Wang, Xiaofang Liu, Shengwang Han, Pengyu Zhu

**Affiliations:** ^1^The Second Affiliated Hospital of Heilongjiang University of Chinese Medicine, No. 411, The Street of Guogeli, Harbin 150001, Heilongjiang, China; ^2^College of Integrated Traditional Chinese and Western Medicine, Hebei Medical University of Chinese Medicine, No. 326, The South of Xinshi Street, Shijiazhuang 050091, Hebei, China

## Abstract

**Background:**

Scalp acupuncture is a contemporary acupuncture method based on the fundamental theories of traditional acupuncture, which has been widely used in patients with stroke in China. However, the effectiveness is controversial due to lack of solid experimental evidence.

**Methods:**

In this study, a rat model of cerebral ischemia-reperfusion injury (CIRI) was established by the middle cerebral artery occlusion/recirculation. The efficacy of scalp acupuncture against CIRI was evaluated by the mNSS scores, TTC staining for brain slices, and laser Doppler perfusion imaging. Immunohistochemical staining for angiogenetic factors indicated the vascularization after CIRI, including VEGF, Ang2, and bFGF. Activation of the Wnt/*β*-catenin signaling pathway and p-GSK3*β* (ser9)/VEGF pathway in the injured brain tissues was assessed by western blotting and qRT-PCR.

**Results:**

On the 7, 14, and 21 days after CIRI, scalp acupuncture could reduce the mNSS scores, decrease the cerebral infarction area, and accelerate the recirculation of ischemic brain tissues. VEGF, FLK1, bFGF, and Ang2 were upregulated on both the mRNA and protein levels in the ischemic brain tissues of the AC group, suggesting that the recirculation might result from angiogenesis, which was also confirmed with the IHC staining in the angiogenetic markers of VEGF, Ang2, and bFGF. Moreover, Wnt3a, *β*-catenin, and cyclin D1 were also upregulated on both the mRNA and protein levels in the ischemic brain tissues of the AC group on day 7, 14, and 21, indicating that the Wnt/*β*-catenin signaling pathway was activated after the treatment of scalp acupuncture. In contrast, dikkoppf-1 (DKK1) pretreatment, a specific inhibitor for the Wnt/*β*-catenin signaling pathway, inactivated the Wnt3a/*β*-catenin signaling pathway and exacerbated the infarct size induced by the cerebral IR injury on day 7.

**Conclusion:**

Together, our findings demonstrated a mechanism whereby scalp acupuncture led to the activation of Wnt/*β*-catenin signaling pathway, promoting angiogenetic factor expression and restoring blood perfusion in the ischemic zone.

## 1. Introduction

Stroke is defined as the sudden death of brain cells due to vascular accidents caused by blockage or rupture of an artery to the brain. As the second leading cause of death and the third leading cause of disability, stroke puts a heavy burden on worldwide socioeconomic development [1, 2]. The primary treatment goal for ischemic stroke is to restore blood flow to the brain regions that are ischemic but not yet infarcted. Hence, early thrombolytic therapy is crucial [3]. tPA is the only FDA-approved medicine for acute ischemic stroke, which restores blood flow to the part being deprived of blood within 4.5 hours of stroke onset [4]. The therapeutic time window limits its application to all stroke patients. Besides, anticoagulant agents can reduce the risk of recurrence and mortality and some agents that can promote vascular remodeling, such as revascularization, angiogenesis, and collateral circle, have been proved to be candidates for the recovery of nerve function [5]. The reality is that those candidates usually fail in clinical trials [6]. The lack of effective medicines and authentication approaches is of critical importance in the clinic [7]. Acupuncture is one of the most widely known Chinese medicine around the world. Scalp acupuncture is based on traditional acupuncture and is widely used for the treatment of ischemic stroke, especially when combined with electrotherapy. Systematic reviews of randomized clinical trials demonstrate that acupuncture improves outcomes in acute stroke and poststroke impairment [8–11]. Accumulating experimental evidence shows that acupuncture exerts protective effects by increasing cerebral blood flow, attenuating ROS damage, maintaining blood-brain barrier integrity, promoting neurodegeneration, and inhibiting cerebral inflammation [11, 12]. It is reported that cerebral ischemia/reperfusion injury (IR) compromises the blood-brain barrier (BBB) and recruits inflammatory cells [13, 14]. The recent studies reveal that the Wnt/*β*-catenin signaling pathway plays important roles in promoting endothelial cell proliferation and maintaining blood-brain barrier homeostasis and has a synergistic effect with the GSK3*β*/VEGF pathway [15]. However, whether electroacupuncture attenuates cerebral IR injury through activating the Wnt/*β*-catenin signaling pathway has not been reported.

In this study, using a CIRI model in rats, we demonstrated that the early application of scalp electroacupuncture on Baihui (GV20) protected the brain tissues from cerebral IR injury [16, 17]. We revealed that scalp electroacupuncture reduced the infarcted areas and restored the blood flow to the ischemic region after CIRI by activating the Wnt/*β*-catenin signaling pathway.

## 2. Materials and Methods

### 2.1. Animals

A total of 100 male Wistar rats (260 ± 20 g) were provided by the Experimental Center of Heilongjiang University of Traditional Chinese Medicine. The rats were randomly divided into five equal groups (*n* = 20 per group): sham operation group (sham), model control group (model), scalp acupuncture group (AC), inhibitor group (DKK1), and scalp acupuncture plus inhibitor group (AC + DKK1). The food and water prepared by the animal center were kept in cages and maintained a normal 12-hour cycle of day and night. The experimental procedures and animal welfare were conducted with approval from the Experimental Center of Heilongjiang University of Traditional Chinese Medicine.

### 2.2. Rat Model of Cerebral Ischemia-Reperfusion Injury (CIRI)

According to the thread-embolism method reported by Zea-Longa [18], all rats underwent CIRI except rats in the sham group. A 3-0 single nylon thread (0.26–0.28 mm diameter), heated one end of the nylon wire to a smooth spherical surface, was marked at the distance of 18 mm from the spherical surface, cleaned by 75% alcohol, and then placed in normal saline for use. Rats were anesthetized by an intra-abdominal injection of 3% pentobarbital (30 mg/kg.b.w) after fasting for 24 hours and weighing. Anesthetized rats were fixed on the operating table. After routine skin preparation disinfection, the neck median was opened with a pair of scissors, and iris forceps was used to carefully dissect the common carotid artery (CCA) free from the vagus nerves without causing physical injury, and common carotid artery (CCA), internal carotid artery (ICA), and external carotid artery (ECA) were exposed. The trunk of the ICA was separated from the pterygopalatine artery (PPA), and an artery clamp was arranged at the starting position and the proximal part of the CCA. The ECA stump was gently pulled down, the 0.2 mm orifice was cut, and the tail end of the nylon line was gently pushed through the CCA bifurcation along the ICA into the skull to reach the middle cerebral artery (MCA). When passed through the bifurcation of MCA and PPA, the direction of the thread bolt was adjusted so that it could enter the MCA smoothly. The insertion depth of the nylon line was about 18 mm by CCA bifurcation. The nylon line was pulled out after 2 hours of embolism, and the blood flow was reperfused. The neurological dysfunction score was used to determine whether the model was successful or not.

### 2.3. Scalp Electroacupuncture Treatment

According to the Laboratory Animal Acupuncture Atlas developed by the National Acupuncture Society for Experimental Research, Baihui (GV20) is located overhead between the middle ears. After successful model establishment, in the acupuncture treatment group, Baihui (GV20) was identified and a circle was drawn with a diameter of 1 cm with Baihui point as the center. Five acupuncture needles (Φ0.5 × 0.16 mm) were used to pierce the five equal points around the circle, and the tip of the needles faced the center of the circle. The points were alternately used for electroacupuncture with a continuous wave of frequency 3–15 Hz and intensity 2–4 mA (Xinsheng electroacupuncture apparatus (G6805-II) produced in Qingdao). Rats received one 30-minute session per day, 7 days for a course, and the brain tissue was collected, respectively, after 1, 2, and 3 courses.

### 2.4. Modified Neurological Severity Score (mNSS)

The motor function of rats was evaluated by Modified Neurological Severity Score (mNSS). The score of the exercise test scale ranged from 0 to 14. Lower scores indicate better neurological function [19]. The rats in each group were tested and recorded by the mNSS test at 7, 14, and 21 days after surgery.

### 2.5. Triphenyl Tetrazolium Chloride (TTC) Staining

The volume of cerebral infarction was measured by TTC. Brain tissues in each group were collected at 7, 14, and 21 days after surgery and treatment, frozen for 20 minutes at −20°C, and then cut into 1 mm slices. The slices were immersed in 2% TTC for 30 minutes at 37°C, and pictures were taken to evaluate the infarcted areas. Image-Pro Plus 5.1 software was used to quantify the infarcted areas with an image analysis system.

### 2.6. Laser Doppler Measurements

The regional cerebral blood flow (rCBF) was measured by using the laser Doppler perfusion imaging system both pre- and postoperatively and 7, 14, and 21 days after surgery and treatment. Rats were anesthetized with an intra-abdominal injection of 3% pentobarbital (30 mg/kg.b.w). Then, rats were fixed in the prone position to maintain the state of anesthesia. The blood flow images of the whole area of the brain were collected by using the laser Doppler high-resolution imaging system (Moor Instruments, USA) and stored in the form of two-dimensional images. The ratio of regional cerebral blood flow in the operation group to the sham operation group was calculated by moorLDI laser Doppler imager review V6.0 analysis software to show the rate of regional cerebral blood flow.

### 2.7. Histopathological Analysis

Brain tissues of rats in each group were collected at 7, 14, and 21 days after surgery and treatment. The brain tissues were perfused with 4% paraformaldehyde, embedded in paraffin, and cut into 3–5 *μ*m slices. The brain tissue sections were stained with hematoxylin and eosin (H&E) staining to assess for brain morphological and histopathological changes.

### 2.8. Immunohistochemical Staining of Brain Tissue

The rats were anesthetized with 3% pentobarbital (30 mg/kg.b.w) and intracardially perfused with chilled saline followed by 4% paraformaldehyde through the left ventricular lumen of the heart. The brains of rats were collected and fixed with 4% paraformaldehyde, embedded in paraffin, and cut into 3–5 *μ*m slices for immunohistochemical staining. The brain tissue sections were kept at 60°C for 24 h in an oven and then deparaffinized with xylene, hydrated with an ethanol gradient (100%–70%), restored with a citrate-EDTA antigen retrieval solution (Beyotime, Shanghai, China) for 15 min at 95°C, and washed three times with PBS when cooled at room temperature. The brain tissue sections were blocked with 10% normal goat nonimmune serum at room temperature for 1 h, and the sections were incubated with rabbit anti-human Ang2 (Cat. No. 24613-1-AP), rabbit anti-human VEGF (Cat. No. 19003-1-AP), and rabbit anti-human bFGF (Cat. No. 19003-1-AP) overnight at 4°C. The next day, the sections were rinsed with PBS and incubated with the corresponding secondary antibodies for 15 min at room temperature followed by diaminobenzidine (DAB) and hematoxylin staining, respectively. All the sections were photographed under a light microscope after sealing with neutral gum.

### 2.9. Western Blot

The brain tissues were placed in RIPA lysis and extraction buffer containing 0.1% phenylmethanesulfonyl fluoride (PMSF), then put into a homogenizer to grind into tissue homogenate, and centrifuged for 10 minutes at 12000 rpm at 4°C. Quantitative determination of protein concentration was done by using BCA. Brain homogenate (40 *μ*g) was separated by SDS-PAGE and transferred to the PVDF membrane. The membrane was then blocked for 1 h in Tris-buffered saline with 5% nonfat milk powder and then incubated with primary antibody (Wnt3a, 1 : 500; *β*-catenin, 1 : 500; TCF-4, 1 : 500; cyclin D1, 1 : 500; p-GSK3*β* (ser9), 1 : 500; VEGF, 1 : 500; Ang2, 1 : 500; bFGF, 1 : 500; FLK1, 1 : 500; and *β*-actin, 1 : 1000) overnight at 4°C. The next day, the membrane was washed with TBST and incubated with the appropriate secondary antibody at 37°C for 1 h. The optical density was quantified by using a biological image analysis system, where the value of sham-operated GR was specified as 1.0.

### 2.10. RNA Extraction and RT-qPCR

The expression levels of Wnt3a, *β*-catenin, TCF-4, cyclin D1, p-GSK3*β* (ser9), VEGF, Ang2, bFGF, FLK1, and *β*-actin were detected by RT-qPCR. In brief, total RNA was extracted from brain tissues of rats using a Eastep^®^ Super Total RNA Extraction Kit (Promega, Shanghai, China). RNA concentrations were estimated with a Nanodrop ND-1000 Spectrophotometer, and then the samples were stored at −80°C. According to the manufacturer's instructions, the oligonucleotides (dT)-primed RNA was reverse transcripted using a Transcriptor First Strand cDNA Synthesis Kit (Roche, Switzerland). RT-qPCR reactions were performed in duplicates using the GoTaq^®^ qPCR Master Mix (Promega, Shanghai, China) according to the manufacturer's protocol. Relative gene expression was assessed via the 2^−ΔΔCT^ method, with GAPDH being used for normalization.

### 2.11. Statistical Analysis

The results were presented as mean ± SD. Statistical analysis was performed using GraphPad Prism 7.0 software and SPSS 22.0 statistical software. Two-tailed unpaired *t*-tests were used to compare differences between two groups, and differences among more than two groups were evaluated using one-way ANOVA. If the ANOVA was statistically significant (*P* ≤ 0.05), the LSD test (parameter method) was used for comparative analysis. Otherwise, Dunnett's test (nonparametric method) was used for comparative analysis (^#^*P* < 0.05, ^##^*P* < 0.01, and ^###^*P* < 0.001 vs. the sham group; ^★^*P* < 0.05, ^★★^*P* < 0.01, and ^★★★^*P* < 0.001 vs. the model group; ^▲^*P* < 0.05 and ^▲▲^*P* < 0.01 vs. the DKK1 group;^+^*P* < 0.05 vs. the AC + DKK1 group).

## 3. Results

### 3.1. Effect of Scalp Electroacupuncture on Cerebral Infarction Size and rCBF of the Cerebral Cortex after CIRI

**Figure 1:**
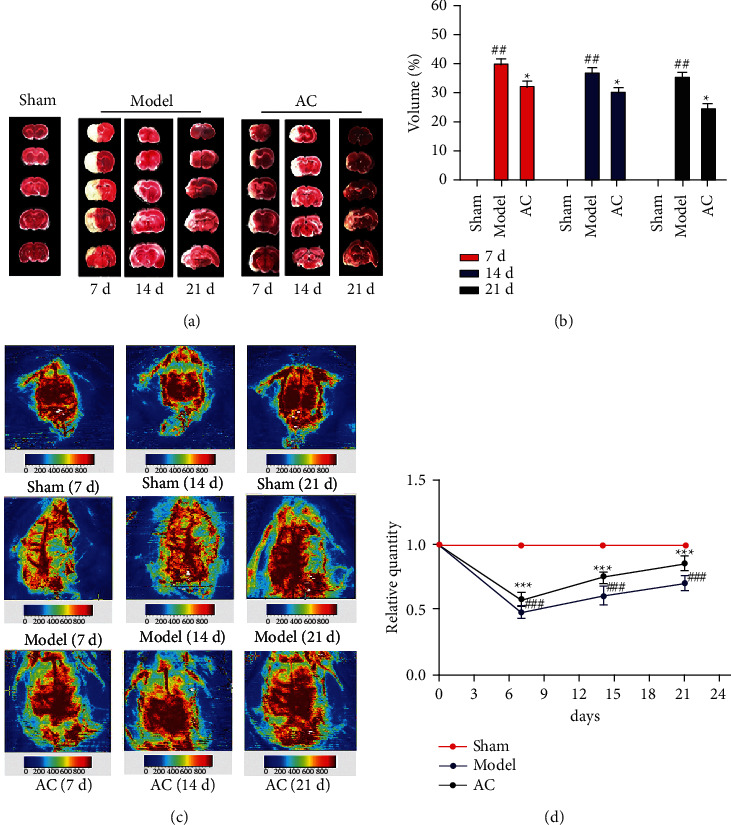
Effect of scalp electroacupuncture on cerebral infarction area and blood flow after cerebral ischemia-reperfusion injury. (a) Cerebral tissues from each group were sectioned into 2-mm slices and then processed for triphenyl tetrazolium chloride (TTC) staining. Normal areas of the brain were stained deep red, and the infarct areas remained unstained. (b) Infarct volume was quantified using the Motic Med 6.0 system and is presented as a percentage of the total brain volume ^##^*P* < 0.01 vs. the sham group; ^★^*P* < 0.05 vs. the model group. (c) Color-coded perfusion images of cerebral cortex were taken by laser Doppler perfusion imaging system. The density of red color represented the tissue perfusion, which can be used to calculate the areas of ischemic cortex. (d) The relative quantity of the areas of ischemic cortex. ^###^*P* < 0.001 vs. the sham group; ^★★★^*P* < 0.001 vs. the model group.

**Figure 2:**
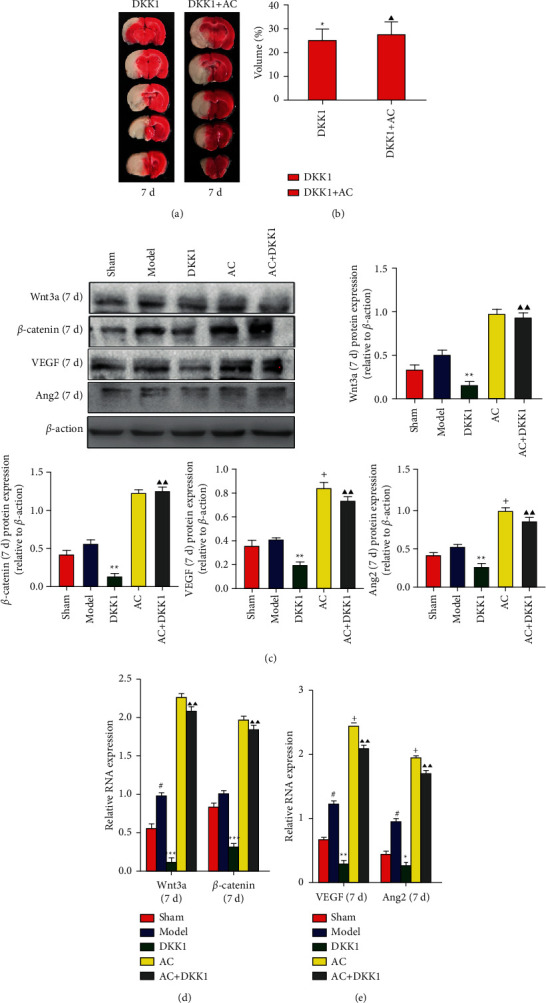
Effect of scalp electroacupuncture on brain tissues after cerebral ischemia-reperfusion injury with DKK1. (a) Cerebral tissues from each group were sectioned into 2-mm slices and then processed for triphenyl tetrazolium chloride (TTC) staining. Normal areas of the brain were stained deep red, and the infarct areas remained unstained. (b) The volume of cerebral infarction was shown. ^★^*P* < 0.05 vs. the model group; ^▲^*P* < 0.05 vs. the DKK1 group. (c) The representative results of western blots together with quantitative analyses in the expression level of Wnt3a, *β*-catenin, VEGF, and Ang2 in the sham, model, DKK1, AC, and AC + DKK1 groups on day 7 postinjury. ^★★^*P* < 0.01 vs. the model group; ^+^*P* < 0.05 vs. the AC + DKK1 group; ^▲▲^*P* < 0.01 vs. the DKK1 group. (d) mRNA expression levels of Wnt3a and *β*-catenin in the sham, model, DKK1, AC, and AC + DKK1 groups on day 7 postinjury. ^★★★^*P* < 0.001, ^★★^*P* < 0.01, and ^★^*P* < 0.05 vs. the model group. (e) mRNA expression levels of VEGF and Ang2 in the sham, model, DKK1, AC, and AC + DKK1 groups on day 7 postinjury. ^#^*P* < 0.05 vs. the sham group; ^★^*P* < 0.05, ^★★^*P* < 0.01, and ^★★★^*P* < 0.001 vs. the model group; ^+^*P* < 0.05 vs. the AC + DKK1 group; ^▲▲^*P* < 0.01 vs. the DKK1 group.

The TTC staining and the rCBF of the cerebral cortex were used to measure the cerebral infarction area and the cerebral blood flow perfusion. The results of TTC staining (Figures 1(a) and 1(b)) showed that the volume of cerebral infarction induced by ischemia-reperfusion injury was significantly increased in the rats of the model group compared with that of the sham group (*P* < 0.01). The volume of cerebral infarction in the AC group was significantly lower than that in the model group (*P* < 0.05). As shown in Figures 2(a) and 2(b), the volume of cerebral infarction in the DKK1 group was significantly lower than that in the model group (*P* < 0.01), and the volume of cerebral infarction in the AC + DKK1 group increased compared with that in the DKK1 group (*P* < 0.05) on day 7. However, there was no difference in the volume of cerebral infarction between the AC and AC + DKK1 groups on day 7.

As shown in the pictures of rCBF Figures 1(c) and 1(d), the baseline data of rCBF were obtained in the normal group. After CIRI, the rCBF dramatically decreased on day 7 (*P* < 0.001) and had recovered on days 14 and 21, but still less than the sham group. Compared with the model group, the rCBF significantly increased at all time points (*P* < 0.001).

### 3.2. Effect of Scalp Electroacupuncture on mNSS Score of Neurological Function and Morphological Changes after CIRI

**Figure 3:**
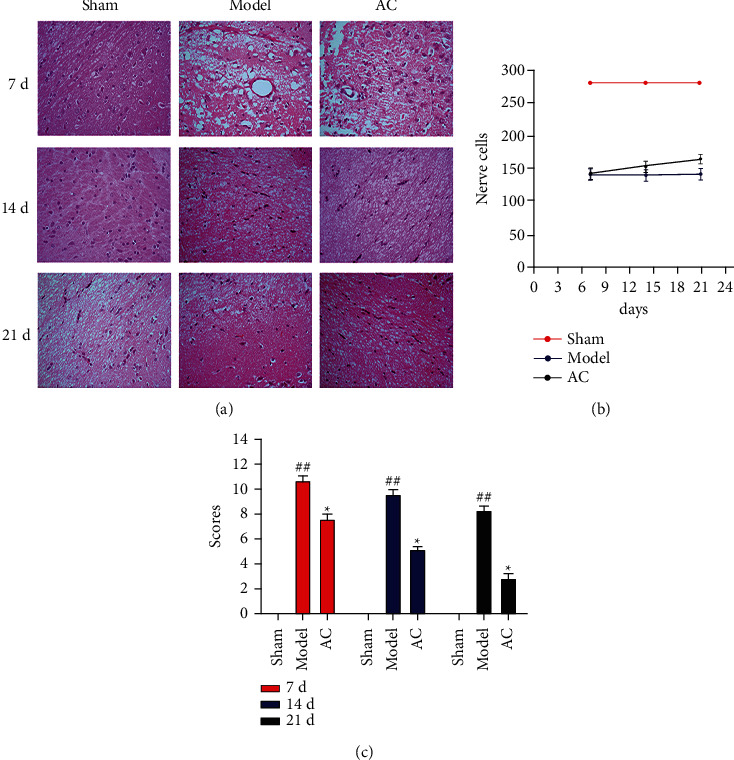
Effects of scalp electroacupuncture on neural protection and function after CIRI. (a) Representative H&E stained images of brain tissue sections (original magnification ×400). (b) Distribution of nerve cells and capillaries. ^###^*P* < 0.001 vs. the sham group; ^★★★^*P* < 0.001 vs. the model group. (c) The mNSS scores were used to assess the neurological function of rats, with lower score representing better neurological function. ^#^*P* < 0.05 and ^##^*P* < 0.01 vs. the sham group; ^★^*P* < 0.05 vs. the model group.

The H&E staining of brain tissues in the sham group showed normal morphological features and no inflammatory cell infiltration. In contrast, the brain tissue sections in the model and AC groups demonstrated the destruction of organizational structures (Figure 3(a)). In the model group, on day 7 of postinjury, the brain tissues showed tissue loss, internal structure derangement, unclear arrangement, swelling of surrounding tissues, nucleus dissolution, and distortion of partial capillaries. All these pathological features got improved on days 14 and 21 of postinjury. Compared with the model group, the pathological features of the AC group were less severe. As shown in Figure 3(b), a semiquantitative analysis for the number of nerve cells indicated that scalp acupuncture showed protective effects against the nerve cell losses on days 7, 14, and 21 of postinjury. As shown in Figure 3(c), compared with the sham group, the mNSS score of the model group increased at all time points (*P* < 0.01). The mNSS score decreased in the AC group than in the model group (*P* < 0.05). The fast recovery motor function was consistent with the protective action of scalp electroacupuncture on the nerve cells in the rats after CIRI.

### 3.3. Protein and mRNA Expression Levels of Wnt3a, *β*-Catenin, TCF4, Cyclin D1, and p-GSK3*β* (ser9)

#### 3.3.1. Protein Expression Levels of Wnt3a, *β*-Catenin, TCF4, Cyclin D1, and p-GSK3*β* (ser9)

**Figure 4:**
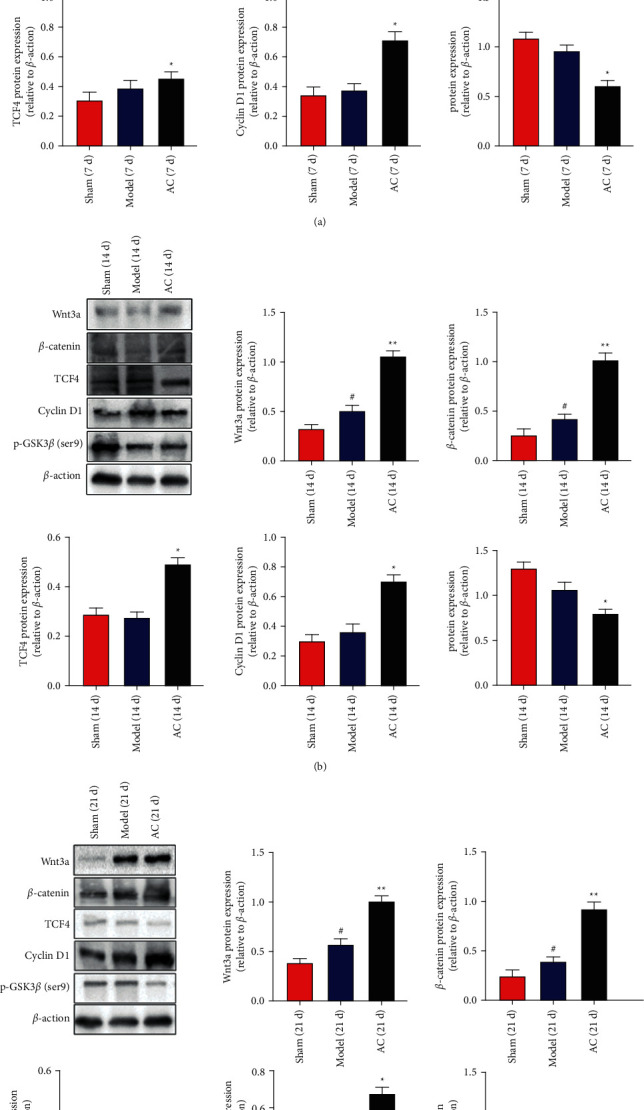
Effects of scalp electroacupuncture on the activation of Wnt/*β*-catenin signaling pathway after CIRI. (a–c) The representative results of western blots together with quantitative analyses in the expression level of Wnt3a, *β*-catenin, TCF4, cyclin D1, and p-GSK3*β* (ser9) in the sham, model, and AC groups on days 7, 14, and 21 postinjury, respectively. ^#^*P* < 0.05 vs. the sham group; ^★^*P* < 0.05 and ^★★★^*P* < 0.001 vs. the model group.

The results of western blotting are shown in Figures 4(a)–4(c), representing the protein expression levels in brain tissues on days 7, 14, and 21 after surgery, respectively. Wnt3a and *β*-catenin expression levels were elevated in the model group from brain tissues compared with those from the sham group (*P* < 0.05) on days 7, 14, 21. However, TCF4 and cyclin D1 expression levels were not different between the model and the sham group on days 7, 14, and 21. The treatment of scalp acupuncture showed increased expression of Wnt3a, *β*-catenin, TCF4, and cyclin D1, compared with the model group on days 7, 14, and 21 (*P* < 0.05 or *P* < 0.01). Moreover, p-GSK3*β* (ser9) expression level was downregulated in the rat brain tissue from the AC group compared with the model group (*P* < 0.05).

To validate the activation of Wnt3a/*β*-catenin pathway, we also detected the expression levels of Wnt3a and *β*-catenin after treating rats with DKK1, a specific inhibitor of Wnt3a, for 7 days. As shown in Figure 2(c), compared with the model group, the expression levels of Wnt3a and *β*-catenin deceased in the brain tissues from the group of DKK1 on day 7 (*P* < 0.01). Wnt3a and *β*-catenin expression levels were increased in the AC + DKK1 group compared with the DKK1 group (*P* < 0.01) on day 7. However, there was no difference in the expression levels of Wnt3a and *β*-catenin between the AC and AC + DKK1 groups on day 7.

#### 3.3.2. mRNA Expression Levels of Wnt3a, *β*-Catenin, TCF4, Cyclin D1, and p-GSK3*β* (ser9)

**Figure 5:**
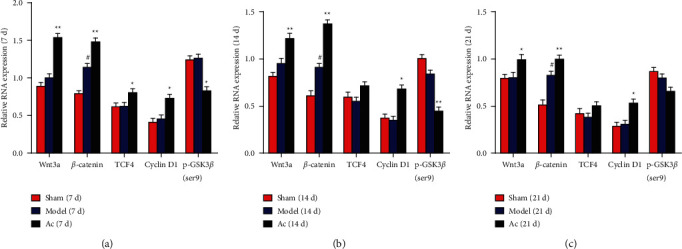
Effects of scalp electroacupuncture on the activation of Wnt/*β*-catenin signaling pathway after CIRI. (a–c) mRNA expression levels of Wnt3a, *β*-catenin, TCF4, cyclin D1, and p-GSK3*β* (ser9) in the sham, model, and AC groups on days 7, 14, and 21 postinjury, respectively. ^#^*P* < 0.05 vs. the sham group; ^★^*P* < 0.05 and ^★★^*P* < 0.01 vs. the model group.

As shown in Figures 5(a)–5(c), consistent with the expression levels of proteins, scalp acupuncture increased the mRNA expression levels of Wnt3a and *β*-catenin in the brain tissue from the AC group compared with that from the model group on days 7, 14, and 21 (*P* < 0.05 or *P* < 0.01). Moreover, compared with the model group, both TCF4 and cyclin D1 mRNA levels increased on day 7 (*P* < 0.05) and only cyclin D1 increased on days 14 and 21. Lastly, we found p-GSK3*β* (ser9) mRNA levels significantly decreased in the AC group compared with the model group on days 7 and 14 (*P* < 0.05).

Similar to the protein assay, we also detected mRNA levels of Wnt3a and *β*-catenin by RT-PCR after adding DKK1. As shown in Figure 2(d), on day 7, mRNA levels of Wnt3a and *β*-catenin were reduced in the DKK1 group compared with the model group (*P* < 0.001). Compared with the DKK1 group, mRNA levels of Wnt3a and *β*-catenin significantly increased in the AC + DKK1 group on day 7 (*P* < 0.01). However, there was no difference in the mRNA levels of Wnt3a and *β*-catenin between the AC and AC + DKK1 groups on day 7.

### 3.4. Immunohistochemical (IHC) Staining of VEGF, Ang2, and bFGF

**Figure 6:**
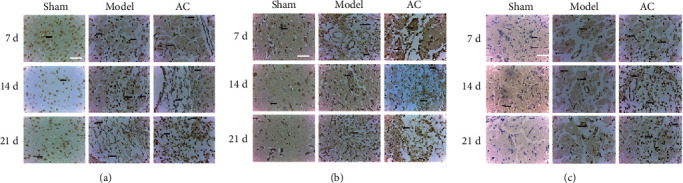
IHC assay for the effect of scalp electroacupuncture on angiogenesis after CIRI. (a–c) Representative immunohistochemical staining for VEGF, Ang2, and bFGF, respectively (original magnification ×400). Black arrows indicate positive cells.

In an IHC assay, proangiogenic factors such as VEGF, Ang2, and bFGF were labeled and observed in the brain tissue sections at each time points (Figure 6). Compared with the sham group, the positive staining of VEGF, Ang2, and bFGF showed a compensatory increase in the model group and a significant increase in the brain tissue from the AC group reaching maximum expression on day 21.

### 3.5. Protein and mRNA Expression Levels of VEGF, FLK1, bFGF, and Ang2

#### 3.5.1. Protein Expression Levels of VEGF, FLK1, bFGF, and Ang2

**Figure 7:**
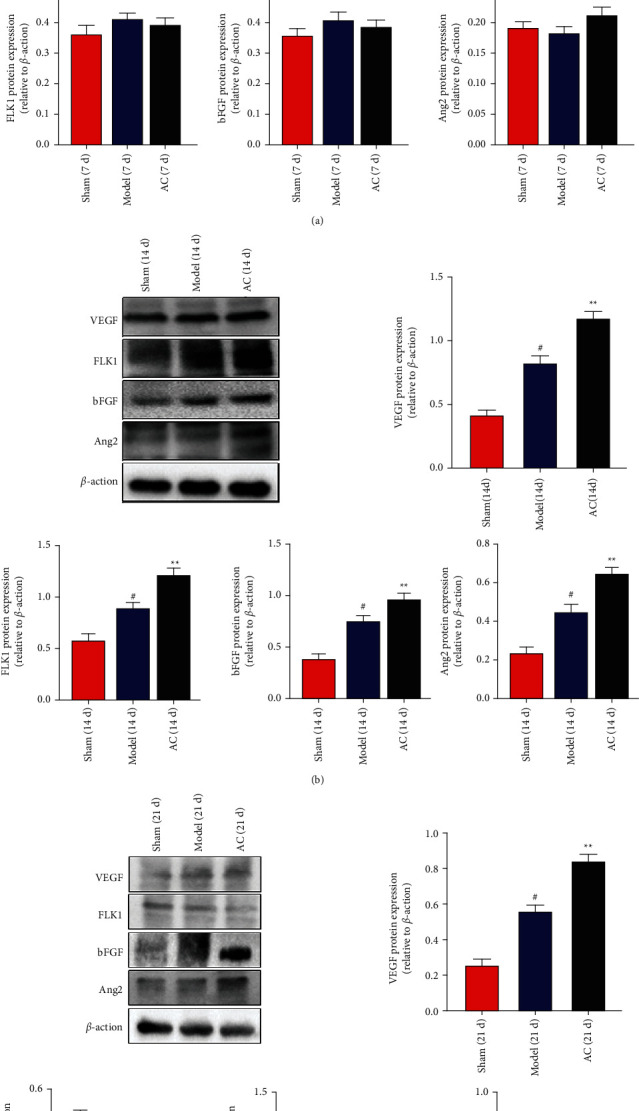
Effects of scalp electroacupuncture on the expression level of the proangiogenic proteins after CIRI. (a–c) The representative results of western blots together with quantitative analyses in the expression level of VEGF, FLK1, bFGF, and Ang2 in the sham, model, and AC groups on days 7, 14, and 21 postinjury, respectively. ^#^*P* < 0.05 vs. the sham group; ^★^*P* < 0.05 and ^★★^*P* < 0.01 vs. the model group.

The results of western blotting are shown in Figures 7(a)–7(c), representing the protein expression levels in brain tissues on days 7, 14, and 21 after surgery, respectively. VEGF, bFGF, and Ang2 expression levels were elevated in the model group from brain tissues compared with those from the sham group (*P* < 0.05) on days 14 and 21. However, VEGF, FLK1, bFGF, and Ang2 expression levels were not different between the model and sham groups on day 7. The treatment of acupuncture showed increased expression of VEGF, FLK1, bFGF, and Ang2 compared with the model group on days 7, 14, and 21 (*P* < 0.05 or *P* < 0.01).

To validate the expression level of the proangiogenic proteins after CIRI, we also detected the expression levels of VEGF and Ang2 after treating rats with DKK1. As shown in Figure 2(c), on day 7, compared with the model group, the expression levels of VEGF and Ang2 deceased in the brain tissues from the group of DKK1 (*P* < 0.05 or *P* < 0.01). VEGF and Ang2 expression levels were increased in the AC + DKK1 group compared with the DKK1 group (*P* < 0.01) on day 7. Compared with the AC + DKK1 group, VEGF and Ang2 expression levels were increased in the AC group (*P* < 0.05) on day 7.

#### 3.5.2. mRNA Expression Levels of VEGF, FLK1, bFGF, and Ang2

**Figure 8:**
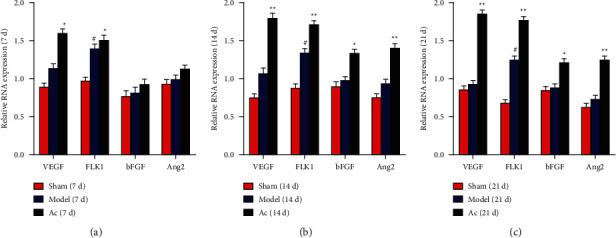
Effects of scalp electroacupuncture on the expression level of the proangiogenic mRNAs after CIRI. (a–c) mRNA expression levels of VEGF, FLK1, bFGF, and Ang2 in the sham, model, and AC groups on days 7, 14, and 21 postinjury, respectively. ^#^*P* < 0.05 vs. the sham group; ^★^*P* < 0.05 and ^★★^*P* < 0.01 vs. the model group.

As shown in Figures 8(a)–8(c), consistent with the expression levels of proteins, scalp acupuncture increased the mRNA expression levels of VEGF and FLK1 in the brain tissue from the AC group compared with that from the model group on days 7, 14, and 21 (*P* < 0.05 or *P* < 0.01). Moreover, compared with the model group, both bFGF and Ang2 mRNA levels increased on days 14 and 21 (*P* < 0.05 or *P* < 0.01).

Similar to the protein assay, we also detected mRNA levels of VEGF and Ang2 by RT-PCR after adding DKK1. As shown in Figure 2(e), on day 7, mRNA levels of VEGF and Ang2 were reduced in the DKK1 group compared with the model group (*P* < 0.05 or *P* < 0.01). VEGF and Ang2 mRNA levels were increased in the AC + DKK1 group compared with the DKK1 group (*P* < 0.01) on day 7. Compared with the AC + DKK1 group, VEGF and Ang2 expression levels increased in the AC group (*P* < 0.05) on day 7.

## 4. Discussion

Accumulating clinical and laboratory evidence has shown that acupuncture therapy can promote neurogenesis, angiogenesis, and neuroplasticity after cerebral ischemia [20]. In clinical practice, a treatment for stroke with the scalp electroacupuncture on Baihui (GV20) has been widely adopted [21, 22]. In this study, the scalp electroacupuncture on GV20 was applied for stroke induced by cerebral IR in rats. The results demonstrated that scalp electroacupuncture could reduce the infarcted areas and restore the blood flow to the ischemic zone, which seemingly increased in a time-dependent way (Figure 1). Moreover, western blotting results revealed that scalp electroacupuncture treatment activated the Wnt/*β*-catenin signaling pathway and elevated angiogenic factors in injured brain tissues, including VEGF, FLK1, bFGF, and Ang2 (Figure 7).

**Figure 9:**
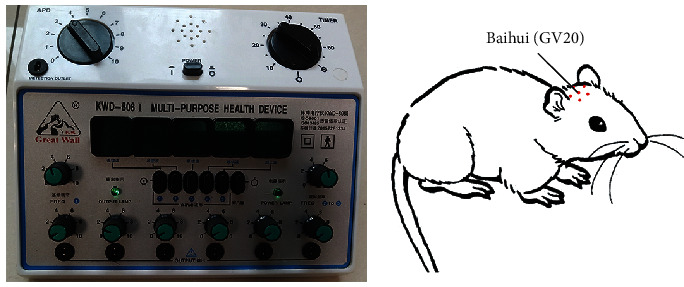
Location of the acupoints and electroacupuncture apparatus.

The core of treatment for stroke is to restore the blood supply to the ischemic area. Early thrombolysis is to restore blood flow and reduce as much cell death of the ischemic region as possible in the stroke acute phase. On the other hand, the therapeutic aim of stroke in the recovery phase is to revascularize the ischemic region to maintain CNS function. Because of the difference of treatment purposes in both acute and chronic phases, the therapeutic regimen of scalp electroacupuncture is shown in Figure 9, and a consecutive treatment was conducted from the very beginning of 30 min after reperfusion to the end of 21 days. Our result showed that improved brain function could be attained on day 7 (acute phase), using an assay of motor functions (mNSS), and the improvement of motor function seemed sustainable with time through mNSS on days 14 and 21 (Figure 1). The fast recovery motor function was consistent with the cerebral histomorphological manifestations of H&E staining, including the decrease of nucleus dissolution and the increases in the number of nerve and capillary cells in the rats of electroacupuncture group (Figure 3). To quantify the size of infarcted area *in vivo*, laser Doppler perfusion images of the cortex were taken at each point in time. Compared with the untreated rats, the early application of scalp electroacupuncture was useful to the restoration of blood supply in the ischemic zones.

The recent studies demonstrated that the classical Wnt/*β*-catenin signal transduction pathway played important roles in maintaining BBB homeostasis and promoting vascularization by synergistic effects with angiogenesis-related factors [7, 23, 24]. The Wnt/*β*-catenin signaling pathway consists of extracellular Wnt signaling molecules, transmembrane receptors, *β*-catenin, GSK3*β*, TCF/LEF, etc. When the extracellular Wnt3a is activated in target cells, a large number of *β*-catenin will move from cytoplasm to nucleus, and then *β*-catenin will combine with transcription factor TCF/LEF to activate the activity of TCF4, regulate the expression of cyclin D1, and promote the expression of the angiogenic factor of VEGF [25, 26]. For instance, lithium application promotes neuroplasticity and recovery after stroke in rats, which upregulated the expression of VEGF to promote endothelial cell proliferation and maintain blood-brain barrier homeostasis through PI3K/GSK3*β*-dependent and -independent pathways [23, 27]. Our results showed that the protein and mRNA expression of Wnt3a and *β*-catenin increased in the brain tissues from the model group at all three time points, which might be a built-in antidamage mechanism (Figures 4 and 5). Notably, no significant increases of TCF4 and cyclin D1 were observed, suggesting that the activation of the Wnt/*β*-catenin pathway caused by cerebral IR injury was insufficient to protect brain tissues from cerebral IR injury [28, 29]. After scalp electroacupuncture treatment, the mRNA and protein expression levels of Wnt3a and *β*-catenin rose in the infarct area of brain tissue and further resulted in cascade upregulation of TCF4 and cyclin D1 (Figures 4 and 5). Moreover, the protein and mRNA expression levels of VEGF and bFGF were also increased in the infarct area of brain tissue from the acupuncture treatment group compared with that from the model group on days 7, 14, and 21, respectively (Figures 6–8). The activation of Wnt/*β*-catenin pathway and elevated *β*-catenin proved to promote the VEGF expression in the endothelial cells [30–32], which indicated that scalp electroacupuncture could activate the Wnt/*β*-catenin signaling pathway to promote angiogenesis. In addition to angiogenesis, the permeability of endothelium after angiogenesis is very important for the establishment of effective vascular function. The angiopoietin (ANG) family cooperates with VEGF to maintain the stability and permeability of vascular endothelial cells [33–35]. Our results showed that scalp electroacupuncture could also promote the protein and mRNA expression of Ang2 on days 7, 14, and 21 (Figures 6–8). To verify the target of scalp electroacupuncture, dikkoppf-1 (DKK1), a specific inhibitor for Wnt/*β*-catenin signaling pathway [36, 37], was applied before scalp electroacupuncture treatment, which could exacerbate the infarct size induced by the cerebral IR injury on day 7. Furthermore, DKK1 pretreatment also impaired the expression of Wnt3a, *β*-catenin, and downstreaming proangiogenic factors (Figure 2). Our results revealed that the efficacy of scalp electroacupuncture on Baihui (GV20) might be, at least in part, attributed to the activation of the Wnt/*β*-catenin signaling pathway for the treatment of stroke.

## 5. Conclusions

In this study, a rat stroke model induced by cerebral ischemia-reperfusion injury was established to reinforce the protective effect of scalp electroacupuncture on Baihui (GV20) [38]. Our findings demonstrated a novel mechanism whereby scalp acupuncture led to the activation of Wnt/*β*-catenin signal pathway, promoting angiogenetic factors' expression and restoring blood perfusion in the ischemic zone. All these results provided the experimental evidence and the novel molecular targets of scalp electroacupuncture on Baihui, which reinforced that the early application of scalp electroacupuncture might protect the brain tissues from cerebral IR injury.

## Data Availability

The data used to support the findings of this study are available from the corresponding author upon request.

## Abbreviations

IR: Ischemia/reperfusion injury
CIRI: Cerebral ischemia-reperfusion injury
BBB: Blood-brain barrier
CCA: Common carotid artery
ICA: Internal carotid artery
ECA: External carotid artery
PPA: Pterygopalatine artery
MCA: Middle cerebral artery
mNSS: Modified neurological severity score
TTC: Triphenyl tetrazolium chloride
rCBF: Regional cerebral blood flow
H&E: Hematoxylin and eosin
DKK1: Dikkoppf-1.

## References

[1] Wang X., Shi S.-H., Yao H.-J. (2016). Electroacupuncture at Dazhui (GV14) and Mingmen (GV4) protects against spinal cord injury: the role of the Wnt/beta-catenin signaling pathway. *Neural Regeneration Research*.

[2] Krishnamurthi R. V., Feigin V. L., Forouzanfar M. H. (2013). Global and regional burden of first-ever ischaemic and haemorrhagic stroke during 1990–2010: findings from the Global Burden of Disease Study 2010. *Lancet Global Health*.

[3] Multicenter Acute Stroke Trial--Europe Study Group, Hommel M., Cornu C., Boutitie F., Boissel J. P. (1996). Thrombolytic therapy with streptokinase in acute ischemic stroke. *New England Journal of Medicine*.

[4] Chavez L. M., Huang S. S., MacDonald I., Lin J. G., Lee Y. C., Chen Y. H. (2017). Mechanisms of acupuncture therapy in ischemic stroke rehabilitation: a literature review of basic studies. *International Journal of Molecular Sciences*.

[5] Kapil N., Datta Y. H., Alakbarova N. (2017). Antiplatelet and anticoagulant therapies for prevention of ischemic stroke. *Clinical and Applied Thrombosis/Hemostasis*.

[6] Boese A. C., Le Q.-S. E., Pham D., Hamblin M. H., Lee J.-P. (2018). Neural stem cell therapy for subacute and chronic ischemic stroke. *Stem Cell Research and Therapy*.

[7] Meffre D., Grenier J., Bernard S. (2014). Wnt and lithium: a common destiny in the therapy of nervous system pathologies?. *Cellular and Molecular Life Sciences*.

[8] Chang Q. Y., Lin Y. W., Hsieh C. L. (2018). Acupuncture and neuroregeneration in ischemic stroke. *Neural Regeneration Research*.

[9] Xu M., Li D., Zhang S. (2018). Acupuncture for acute stroke. *Cochrane Database of Systematic Reviews*.

[10] Wang J., Pei J., Khiati D. (2017). Acupuncture treatment on the motor area of the scalp for motor dysfunction in patients with ischemic stroke: study protocol for a randomized controlled trial. *Trials*.

[11] Tian L., Wang J.-H., Sun R.-J., Zhang X.-H., Yuan B.-O., Du X.-Z. (2016). Development of researches on scalp acupuncture for ischemic stroke. *Acupuncture Research*.

[12] Zhou J.-W., Li J., Zhao J.-J., Xie H.-J., Wang M. (2013). Meta analysis on ischemic stroke treated with scalp acupuncture. *World Journal of Acupuncture—Moxibustion*.

[13] National Institute of Neurological Disorders and Stroke rt-PA Stroke Study Group (1995). Tissue plasminogen activator for acute ischemic stroke. *New England Journal of Medicine*.

[14] Zhou Y., Nathans J. (2014). Gpr124 controls CNS angiogenesis and blood-brain barrier integrity by promoting ligand-specific canonical wnt signaling. *Developmental Cell*.

[15] Wang W., Li M., Wang Y. (2016). GSK-3*β* inhibitor TWS119 attenuates rtPA-induced hemorrhagic transformation and activates the Wnt/*β*-catenin signaling pathway after acute ischemic stroke in rats. *Molecular Neurobiology*.

[16] Young-Wook P., Yoon H. G., Jae K. M., Seo-Yeon L., Tae C. B., Kyoung S. H. (2019). Subacute electroacupuncture at Baihui (GV 20) and Dazhui (GV 14) promotes post-stroke functional recovery via neurogenesis and astrogliosis in a photothrombotic stroke mouse model. *Journal of Traditional Chinese Medicine*.

[17] Zhou F., Guo J., Cheng J., Cheng J., Wu G., Xia Y. (2011). Electroacupuncture increased cerebral blood flow and reduced ischemic brain injury: dependence on stimulation intensity and frequency. *Journal of Applied Physiology*.

[18] Longa E. Z., Weinstein P. R., Carlson S., Cummins R. (1989). Reversible middle cerebral artery occlusion without craniectomy in rats. *Stroke*.

[19] Lei C., Wu B., Cao T., Zhang S., Liu M. (2015). Activation of the high-mobility group box 1 protein-receptor for advanced glycation end-products signaling pathway in rats during neurogenesis after intracerebral hemorrhage. *Stroke*.

[20] Zhu W., Ye Y., Liu Y. (2017). Mechanisms of acupuncture therapy for cerebral ischemia: an evidence-based review of clinical and animal studies on cerebral ischemia. *Journal of Neuroimmune Pharmacology*.

[21] Zhan J., Pan R., Guo Y. (2016). Acupuncture at Baihui (GV 20) and Shenting (GV 24) combined with basic treatment and regular rehabilitation for post-stroke cognitive impairment:a randomized controlled trial. *Chinese Acupuncture and Moxibustion*.

[22] Dong H., Zhao H.-Y., Wang J.-W., Han J.-X. (2019). Observation on therapeutic effect and mechanism research of acupuncture on headache in the recovery phase of ischemic stroke. *Chinese Acupuncture and Moxibustion*.

[23] Du J., Li J. (2018). The role of Wnt signaling pathway in atherosclerosis and its relationship with angiogenesis. *Experimental and Therapeutic Medicine*.

[24] Matthijs Blankesteijn W., Hermans K. C. M. (2015). Wnt signaling in atherosclerosis. *European Journal of Pharmacology*.

[25] Jiang L., Yin M., Wei X. (2015). Bach1 represses wnt/*β*-catenin signaling and angiogenesis. *Circulation Research*.

[26] Xu Y., Zhang G., Kang Z., Xu Y., Jiang W., Zhang S. (2016). Cornin increases angiogenesis and improves functional recovery after stroke via the Ang1/Tie2 axis and the Wnt/*β*-catenin pathway. *Archives of Pharmacal Research*.

[27] Guo S., Arai K., Stins M. F., Chuang D.-M., Lo E. H. (2009). Lithium upregulates vascular endothelial growth factor in brain endothelial cells and astrocytes. *Stroke*.

[28] Mu Q., Zhou H., Xu Y. (2020). NPD1 inhibits excessive autophagy by targeting RNF146 and wnt/*β*-catenin pathway in cerebral ischemia-reperfusion injury. *Journal of Receptors and Signal Transduction Research*.

[29] Pan W., Xu Z. (2020). Triptolide mediates Wnt/*β*-catenin signalling pathway to reduce cerebral ischemia-reperfusion injury in rats. *Folia Neuropathologica*.

[30] Tang E., Wang Y., Liu T., Yan B. (2019). Gastrin promotes angiogenesis by activating HIF-1*α*/*β*-catenin/VEGF signaling in gastric cancer. *Gene*.

[31] Wiström J., Norrby S. R., Burman L. G., Lundholm R., Jellheden B., Englund G. (1987). Norfloxacin versus placebo for prophylaxis against travellers’ diarrhoea. *Journal of Antimicrobial Chemotherapy*.

[32] Skurk C., Maatz H., Rocnik E., Bialik A., Force T., Walsh K. (2005). Glycogen-synthase kinase3*β*/*β*-catenin axis promotes angiogenesis through activation of vascular endothelial growth factor signaling in endothelial cells. *Circulation Research*.

[33] Xu Y.-N., Zhang Z., Ma P., Zhang S.-H. (2011). Adenovirus-delivered angiopoietin 1 accelerates the resolution of inflammation of acute endotoxic lung injury in mice. *Anesthesia and Analgesia*.

[34] Huang Y. Q., Sauthoff H., Herscovici P. (2008). Angiopoietin-1 increases survival and reduces the development of lung edema induced by endotoxin administration in a murine model of acute lung injury. *Critical Care Medicine*.

[35] van der Heijden M., van Nieuw Amerongen G. P., van Bezu J., Paul M. A., Groeneveld A. B. J., van Hinsbergh V. W. M. (2011). Opposing effects of the angiopoietins on the thrombin-induced permeability of human pulmonary microvascular endothelial cells. *PLoS One*.

[36] Scott E. L., Brann D. W. (2013). Estrogen regulation of Dkk1 and Wnt/*β*-Catenin signaling in neurodegenerative disease. *Brain Research*.

[37] Mastroiacovo F., Busceti C. L., Biagioni F. (2009). Induction of the Wnt antagonist, Dickkopf-1, contributes to the development of neuronal death in models of brain focal ischemia. *Journal of Cerebral Blood Flow and Metabolism*.

[38] Mao C., Hu C., Zhou Y. (2020). Electroacupuncture pretreatment against cerebral ischemia/reperfusion injury through mitophagy. *Evidence-Based Complementary and Alternative Medicine*.

